# Supplementary material for: Distinct Colitis-Associated Macrophages Drive NOD2-Dependent Bacterial Sensing and Gut Homeostasis
Source: bioRxiv. 2025 Jul 31:2025.01.21.634180. Preprint. [Version 2] doi: 10.1101/2025.01.21.634180 (PMC12324400; doi:10.1101/2025.01.21.634180)
Supplement: Supplement 1 [file media-1.pdf]

Distinct Colitis-Associated Macrophages Drive NOD2-Dependent Bacterial Sensing and Gut Homeostasis

Authors: Gajanan D. Katkar[1†], Mahitha Shree Anandachar[1,2†], Stella-Rita Ibeawuchi[2], Ella McLaren[1], Megan Estanol[1], Kennith Carpio-Perkins[1], Shu-Ting Hsu[1], Celia R. Espinoza[1], Jane Coates[1], Yashaswat S. Malhotra[1], Madhubanti Mullick[1], Vanessa Castillo[1], Daniella T. Vo[3], Saptarshi Sinha[1], and Pradipta Ghosh[1,4*]

Affiliations:
[1]Department of Cellular and Molecular Medicine, University of California San Diego, CA, USA.
[2]Department of Pathology, University of California San Diego, CA, USA.
[3]Department of Pediatrics, University of California San Diego, CA, USA.
[4]Department of Medicine, University of California San Diego, CA, USA.

[†] Equal contribution

Conflict of interest statement: Authors have declared that no conflict of interest exists.

KEY WORDS: GIV/Girdin, Guanine-nucleotide exchange modulators (GEMs), CCDC88A, Macrophage, NOD2, MDP, Microbes, Innate immunity

***Correspondence to**:

Pradipta Ghosh, M.D.; Professor, Departments of Medicine, and Cell and Molecular Medicine, University of California San Diego; 9500 Gilman Drive (MC 0651), George E. Palade Bldg, Rm 232, 239; La Jolla, CA 92093. Phone: 858-822-7633: Fax: 858-822-7636: Email: prghosh@ucsd.edu

CATALOG OF SUPPLEMENTARY MATERIALS

1. *Supplementary Table (S1)*
2. *Supplementary Figures and Legends (S1-S4)*

SUPPLEMENTARY TABLES

Supplementary Table 1: NOD2 mutations and its clinical relevance in CD patients.

Mutation	Percentage of CD Patients	% CD diagnosis	Mechanistic insights	Variation Caused	Phenotypic Consequence
**1007fs (Frameshift Stop codon mutation)**	31%	100%	Impaired palmitoylation (Lu et al. 2019) , and protein mislocalization<br><br>Forced PM-localization of protein *does not* restore function (Lécine et al. 2007)<br><br>MDP-binding site unaffected<br><br>Loss of binding to GIV (current work)	- Mutant is unable to recognize MDP to initiate NFkB activation<br>- Associated defective release of IL-10 from blood mononuclear cells (PBMC) after stimulation with the TLR2 ligands, PGN and Pam3Cys-KKKK, and LPS.	- The genotype relative risk (GRR) for developing CD in heterozygotes and homozygotes of this mutation alone is 3.29 ±0.64 and 34.66 ±12.87 respectively<br><br>- Homozygosity is strongly associated with gastroduodenal CD and younger age at diagnosis<br><br>- Homozygous patients demonstrate a much more severe disease phenotype than other patients with Crohn's disease and have an increased risk for ileal stenoses and surgical interventions
**R702W (Missense Substitution Mutation)**	32%	100%	Impaired palmitoylation (Lu et al. 2019) , and protein mislocalization<br><br>Forced PM-localization of protein restores function (Lécine et al. 2007)<br><br>MDP-binding site unaffected<br><br>Loss of binding to GIV (current work)	- Monocyte-derived dendritic cells (MoDCs) produced significantly higher levels of IL-12 on stimulation with whole bacteria<br><br>- MoDCs carrying the mutation displayed an increased basal level of IL-8 release, which, after a bacterial encounter, equilibrated to the levels similar to healthy controls.	- GRR for developing CD in heterozygotes and homozygotes of this mutation alone is 1.97±0.85 and roughly 3.05 respectively<br><br>- Positive independent association with structuring behavior and granuloma formation
**G908R (Missense Substitution Mutation)**	18%	80%	Normal palmitoylation (Lu et al. 2019) and protein mislocalization.<br><br>Forced PM-localization of protein restores function (Lécine et al. 2007)<br><br>One of the residues that form contact site for MDP and hence, MDP recognition could be directly impacted (Vijayrajratnam et al. 2017).<br>GIV binding (current work).	-MoDCs produced significantly higher levels of IL-12 on stimulation with whole bacteria	- GRR for developing CD in heterozygotes and homozygotes of this mutation alone is 1.97±0.85 and 4.55±1.34 respectively

SUPPLEMENTARY FIGURES

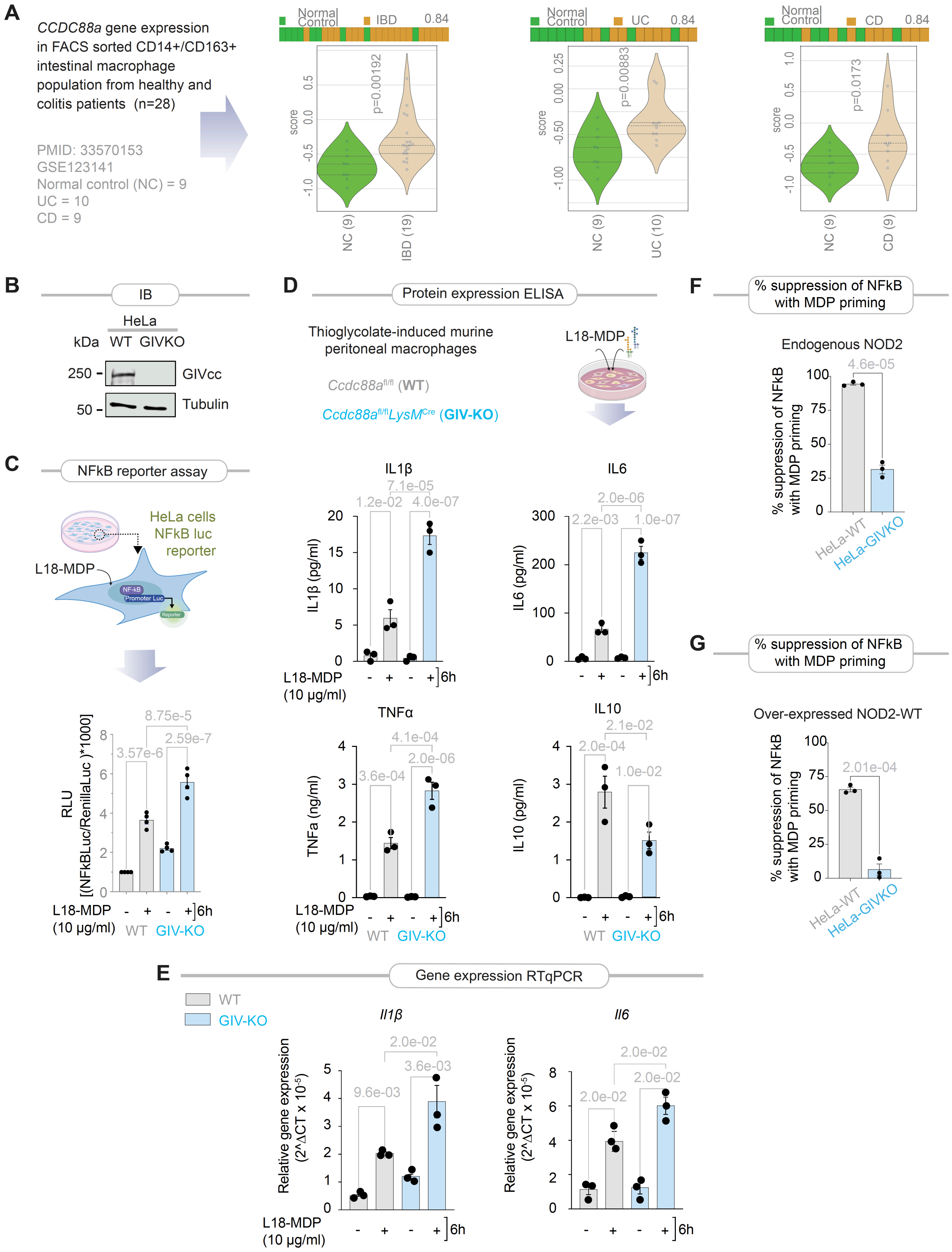

Supplementary Figure 1: GIV is required for MDP-stimulated inflammatory resolution. Related to **Figure 1, 2 and 5**.

A. Violin plots display *CCDC88A* expression in macrophages that were isolated from the lamina propria of colons from healthy and IBD (UC and CD) subjects. *p*-values are calculated using Welch's t-test. Bar plots on top denote sample classification accuracy using *CCDC88A* as single gene expression score and the numbers on top indicate ROC AUC.

B. Immunoblot (IB) of control (HeLa-WT) or GIV-KO (HeLa-GIV-KO) HeLa cells confirming depletion of GIV by >95%.

C. Schematics display the NFkB reporter assay in HeLa cells (*top*) and bar graphs display the fold change in NFkB activity (*bottom*).

D. Schematic (top) of study design to assess cytokines produced by peritoneal macrophage stimulated with L18-MDP (10 µg/ml) for 6 h. Bar graphs (bottom) display the concentrations of the indicated cytokines, as determined by ELISA.

E. Bar graphs display the gene expression in cell lysates in D, as determined by RT qPCR.

F-G. Bar graphs display the fold change in NFkB activity induced by LPS (100 ng/ml) for 6h, following 24-hour priming with L18-MDP (10 µg/ml) in HeLa cells. Panel F represents cells with endogenous NOD2, while Panel G represents cells with overexpressed NOD2.

Statistics: All results are displayed as mean ± SEM (n=3 biological replicates). Statistical significance was tested using two-way/one-way ANOVA followed by Tukey's test for multiple comparisons. *p*-value ≤ 0.05 is considered as significant.

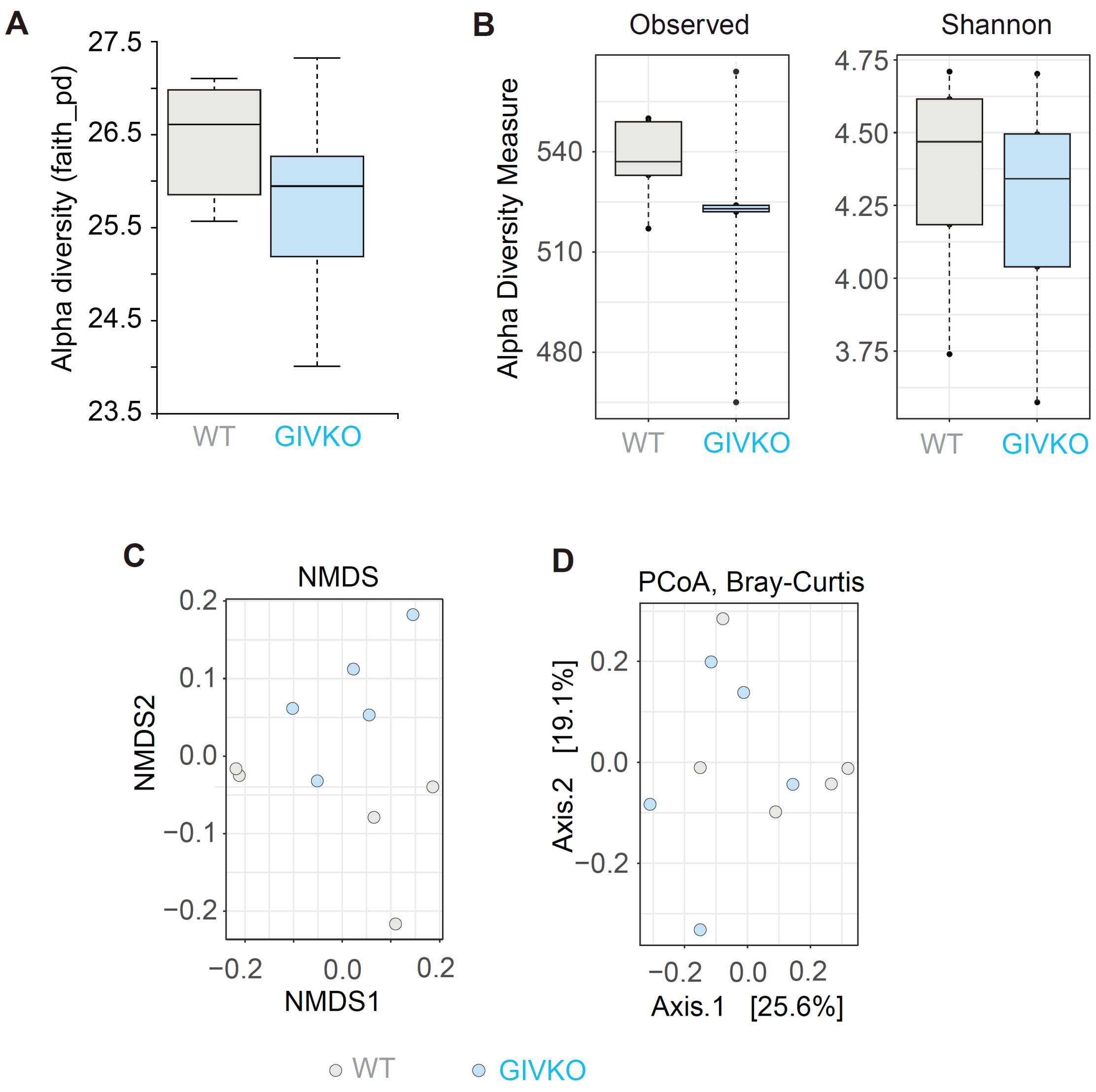

Supplementary Figure 2: Fecal microbiome analysis confirms spontaneous dysbiosis in myeloid-specific (LysMCre) GIV-KO mice age 8-12 wk. Related to **Figure 4A**.

A-B. Box plots display alpha diversity indices (Observed and Shannon), of fecal microbiome communities within GIV-KO and their littermate controls.

C. Non-Metric Multidimensional Scaling (NMDS) ordination of fecal microbiome communities within GIV-KO and their littermate controls

D. PCoA plot of beta diversity of fecal microbiome communities within GIV-KO and their littermate controls.

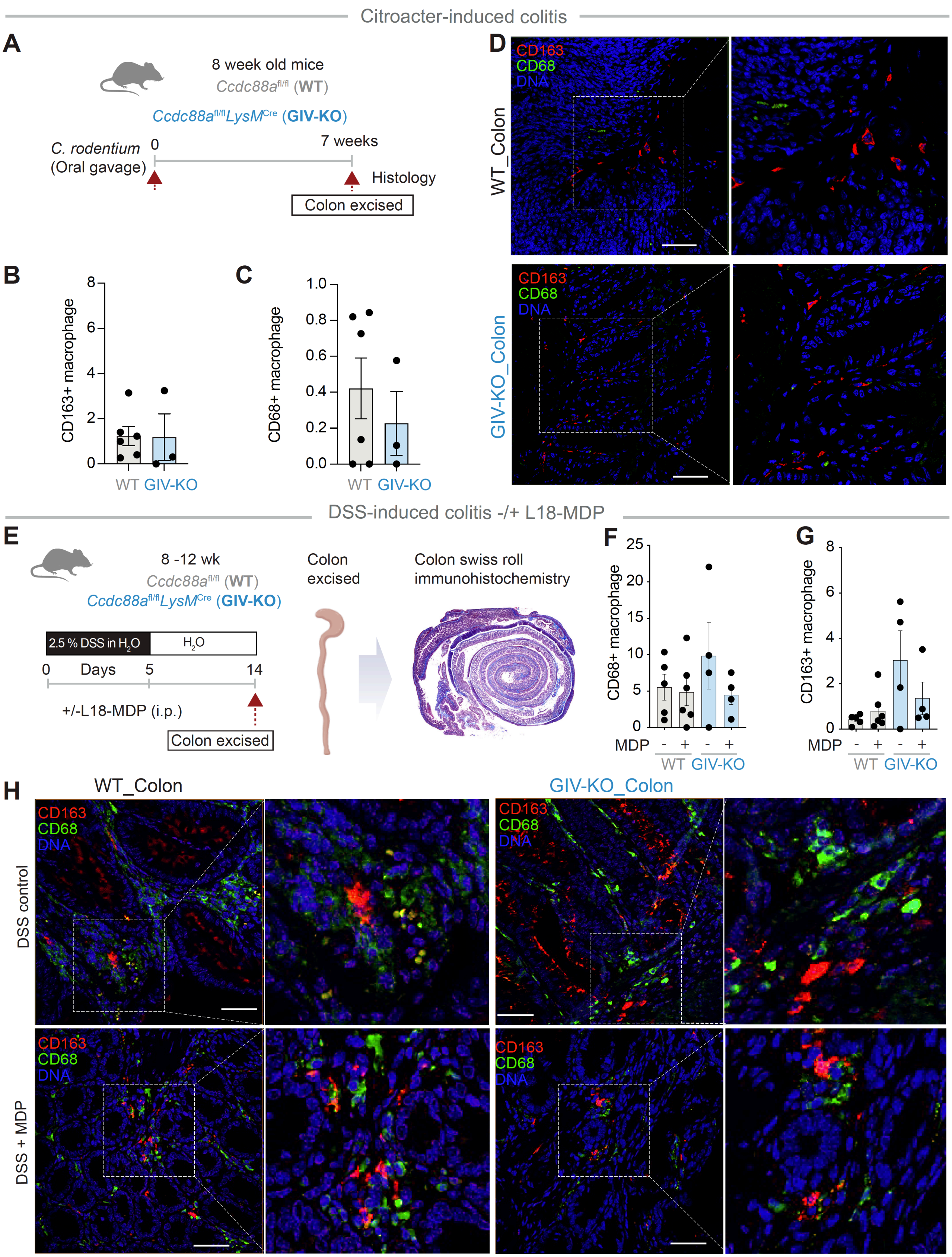

Supplementary Figure 3: The Loss of GIV does not impede macrophage recruitment and expression of conventional markers for polarization. Related to **Figures 4-5**.

A-D. Panels describing the experimental design (**A**) and macrophage population distribution in the colon (**B-D**) in an infectious colitis model of GIV-KO and control littermates induced using *Citrobacter rodentium* (initially

termed *Citrobacter freundii* biotype 4280 (1); strain name DBS100; 5 x 10^8 CFU/200ul/mouse. GIV-KO, n=8; WT, n=6. Findings are representative of two independent repeats. *See Figure 4C-I for the details*.
B-C. Quantification of CD163$^+$ (B) and CD68$^+$ (C) macrophages in colonic sections. n=4-6. **D.** Representative immunofluorescence images showing CD163 (red), CD68 (green), and nuclei (blue) in colon tissues. Scale bars = 50 µm.
E–H. Schematic (E) displays the study design for DSS-induced colitis. GIV-KO, n=5; WT, n=5. Findings are representative of two independent repeats. *See Figure 5A-E for the details.*
(F–G) Quantification of CD68$^+$ (F) and CD163$^+$ (G) macrophages in colon tissues. n = 5–6 mice per group.
(H) Representative immunofluorescence images of colon sections from DSS-only and DSS+MDP groups showing CD163 (red), CD68 (green), and nuclei (blue). Scale bars = 50 µm.
All graphs represent mean ± SEM. Statistical comparisons were made using unpaired t-tests. p-value ≤ 0.05 is considered as significant.

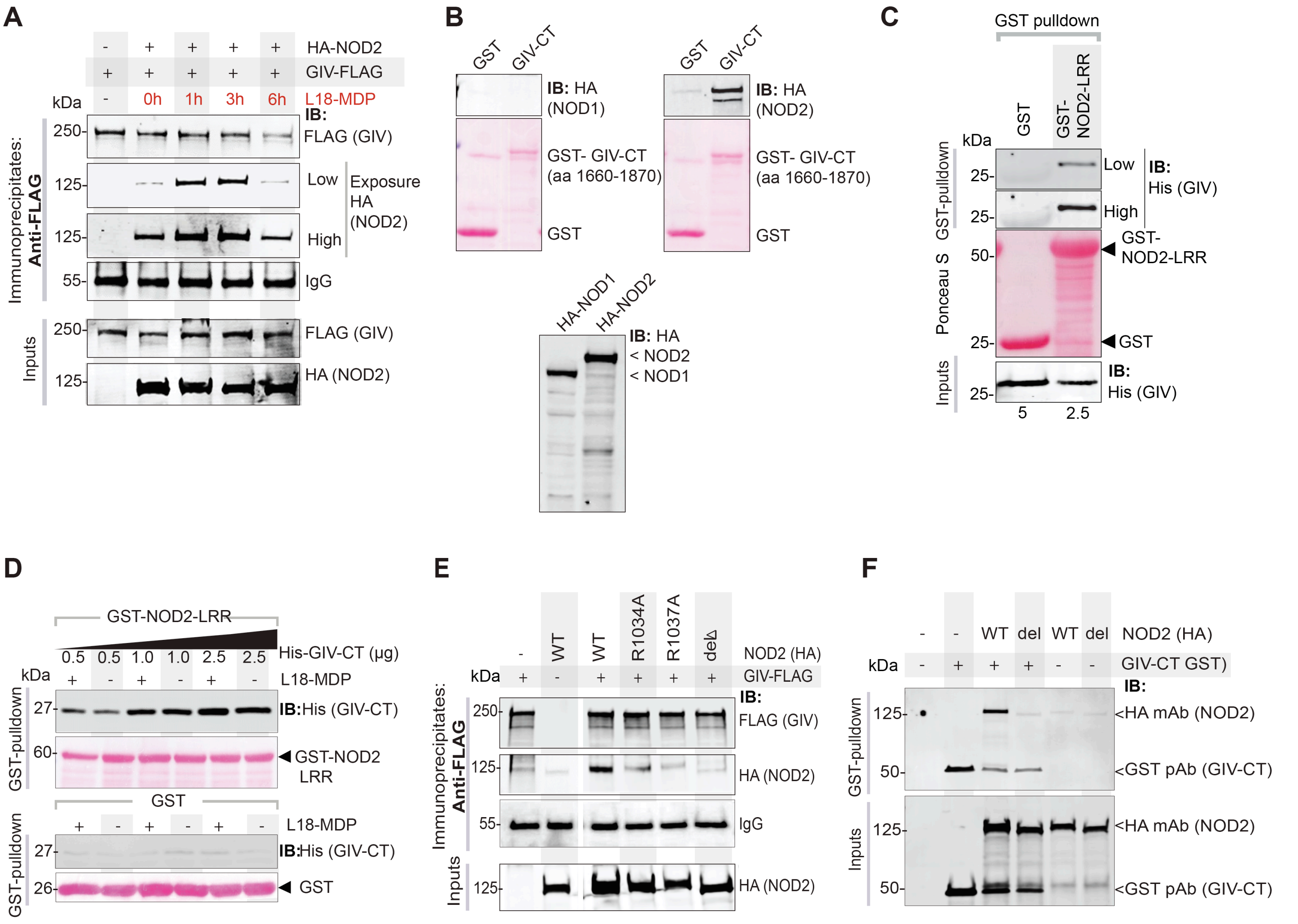

Supplementary Figure 4: The NOD2(LRR)●GIV(C-term) interaction is a dynamic. Related to **Figures 7-8**.

A. FLAG-tagged GIV was immunoprecipitated with anti-FLAG mAb from equal aliquots of lysates of HEK cells expressing GIV-FLAG and HA-NOD2, stimulated (+) or not (-) with L18-MDP (10 μg/ml) for indicated time points. Immunoprecipitated (IP; top) complexes and input (bottom) lysates were analyzed for NOD2 and GIV by immunoblotting (IB).

B. Lysates of HEK cells expressing HA-tagged NOD1/2 proteins were used in a GST pulldown assay with GST or GST-GIV-CT immobilized on Glutathione Sepharose beads. Bound NOD1/2 proteins were visualized by immunoblotting (IB).

C. Recombinant His-GIV-CT proteins were used in a GST pulldown assay with GST or GST-NOD2-LRR immobilized on Glutathione Sepharose beads. Bound GIV were visualized by immunoblotting (IB).

D. Recombinant His-GIV-CT proteins (0.5, 1, 2.5 μg) were used in a GST pulldown assay with GST or GST-NOD2-LRR immobilized on Glutathione Sepharose beads, in presence (+) or absence (-) of 10x molar excess of L18-MDP. If MDP and GIV share the same binding site on the LRR domain of NOD2, excess of MDP is expected to compete with GIV and reduce its binding to NOD2-LRR. Bound GIV was visualized by immunoblotting (IB).

E. FLAG-tagged GIV was immunoprecipitated with anti-FLAG mAb from equal aliquots of lysates of HEK cells expressing GIV-FLAG and either wild-type (WT) or mutant HA-NOD2 constructs. Immunoprecipitated (IP; top) complexes and input (bottom) lysates were analyzed for NOD2 by immunoblotting (IB).

F. GST-GIV-CT was pulled down using Glutathione Sepharose beads from equal aliquots of lysates of HEK lysates expressing the wild-type (WT) or del mutant HA-NOD2 construct either alone (last two lanes) or with GST-GIV-CT (aa 1660-1870; mammalian p-CEFL vector). Bound NOD2 proteins and similar expression of GIV-CT was assessed by immunoblotting (IB) using anti-HA (NOD2) and anti-GST (GIV-CT) antibodies.

Uncategorized References

1. Newman JV, Zabel BA, Jha SS, and Schauer DB. Citrobacter rodentium espB is necessary for signal transduction and for infection of laboratory mice. *Infect Immun.* 1999;67(11):6019-25.
